# The NOAEL Metformin Dose Is Ineffective against Metabolic Disruption Induced by Chronic Cadmium Exposure in Wistar Rats

**DOI:** 10.3390/toxics6030055

**Published:** 2018-09-10

**Authors:** Victor Enrique Sarmiento-Ortega, Eduardo Brambila, José Ángel Flores-Hernández, Alfonso Díaz, Ulises Peña-Rosas, Diana Moroni-González, Violeta Aburto-Luna, Samuel Treviño

**Affiliations:** 1 Laboratory of Chemical-Clinical Investigations, Department of Clinical Chemistry, Faculty of Chemistry Science, University Autonomous of Puebla, 14 South. CQ1, University City, Puebla C.P. 72560, Mexico; qfb_veso111@hotmail.com (V.E.S.-O.); eduardobrambila1@yahoo.com.mx (E.B.); quimicoangel32@hotmail.com (J.Á.F.-H.); d.moroni_25@hotmail.com (D.M.-G.); val_140485@hotmail.com (V.A.-L.); 2 Department of Pharmacy, Faculty of Chemistry Science, University Autonomous of Puebla, 14 South. CQ1, University City, Puebla C.P. 72560, Mexico; dan_alf2005@yahoo.com.mx; 3 Department of Analytic Chemistry, Faculty of Chemistry Science, University Autonomous of Puebla, 14 South. CQ1, University City, Puebla C.P. 72560, Mexico; quim_perua@hotmail.com

**Keywords:** metformin, cadmium toxicity, metabolic disruptor, metabolic syndrome

## Abstract

Previous studies have proposed that cadmium (Cd) is a metabolic disruptor, which is associated with insulin resistance, metabolic syndrome, and diabetes. This metal is not considered by international agencies for the study of metabolic diseases. In this study, we investigate the effect of metformin on Cd-exposed Wistar rats at a lowest-observed-adverse-effect level (LOAEL) dose (32.5 ppm) in drinking water. Metabolic complications in the rats exposed to Cd were dysglycemia, insulin resistance, dyslipidemia, dyslipoproteinemia, and imbalance in triglyceride and glycogen storage in the liver, muscle, heart, kidney, and adipose tissue. Meanwhile, rats treated orally with a No-observable-adverse-effect level (NOAEL) dose of metformin (200 mg/kg/day) showed mild improvement on serum lipids, but not on glucose tolerance; in tissues, glycogen storage was improved, but lipid storage was ineffective. In conclusion, metformin as a first-line pharmacological therapy must take into consideration the origin and duration of metabolic disruption, because in this work the NOAEL dose of metformin (200 mg/kg/day) showed a limited efficiency in the metabolic disruption caused by chronic Cd exposure.

## 1. Introduction

Cadmium (Cd) is a transition metal that represents a health risk, being classified as one of the top five most hazardous environmental contaminants by the Agency for Toxic Substances and Disease Registry [1]. Human exposure to Cd occurs mainly through inhalation or ingestion, and its absorption depends on the particle size, concentration, time-exposure, and competitivity with biometals such as iron, calcium, or zinc. Cigarette smoking is considered to be the most significant source of human exposure to Cd [2,3,4,5]. In humans and other mammals, Cd can damage several organs and tissues, including the kidneys, liver, lung, pancreas, testis, placenta, brain, and bone, but the kidneys and liver are the two primary target organs [5,6,7,8]. Damage to tissues is accompanied by a variable degree of injury because of inflammation and oxidative stress [9,10,11,12,13]. Likewise, Cd is referred to as a heavy metal that causes endocrine disruption [14,15,16], and recently as a metabolic disruptor because it has been described as a risk factor for the developing of insulin resistance, metabolic syndrome, obesity, and diabetes; however, the international agencies for the study of metabolic diseases have not yet considered these issues officially [17,18,19,20].

According to world sanitary statistics in 2014 emitted by the World Health Organization (WHO), there are almost 387 million diagnosed cases of type 2 diabetes mellitus (T2DM), but it is estimated that 178 million remain undiagnosed and this is expected to reach 592 million in 2035, which will contribute to health expenses of approximately $245 billion in the U.S. alone. Correspondingly, diabetes has been intimately linked to obesity and overweight problems, which represent a third of the worldwide population. Because of its extremely high prevalence, obesity has a significant socioeconomic impact of approximately $190 billion/year in the U.S. [21,22,23,24]. Obesity and T2DM belong to a very complex group of genetic and epigenetic diseases with a socio-environmental influence known as chronic non-communicable diseases, that have a common background: metabolic disturbances or metabolic syndrome associated with dysglycemia, dyslipidemia, dyslipoproteinemia, and arterial hypertension, as well as hormone imbalance of insulin, leptin, adiponectin, and resistin, which affect other hormonal axes, contributing to alterations of triglycerides and glycogen in several tissues [25,26,27].

The first line of pharmacological therapy for metabolic disorders is metformin (1,1-dimethyl biguanide) because it can control each complication associated with metabolic syndrome in variable degrees. With approximately 50 years of accumulated global clinical experience, metformin is generally regarded as safe [28]. Metformin has demonstrated its efficiency in lowering blood glucose levels, reducing mild weight problems in people with a high body mass index (BMI), improving insulin sensitivity and insulin secretion, and modulating multiple incretin axis components, all of which have only a minimal risk of hypoglycemia, and regulating triglyceride and cholesterol levels [29,30]. Recently, metformin has been confirmed by the American Diabetes Association and the European Association for the Study of Diabetes as a pharmacological therapy [31,32]. However, the dosage is a sensitive issue because the diabetic patients can consume up to 2000 mg per day, in two to three divided doses. In this sense, the adaptation of therapeutical doses has been studied in animal models, such as rats, in order to understand the toxicological effects. The hypoglycemic effects with a no observable adverse effect level (NOAEL) were 200 mg/kg/day. Meanwhile, a dose of ≥600 mg/kg/day observed adverse findings including an increased incidence of minimal necrosis, inflammation, and metabolic acidosis (increased serum lactate and beta-hydroxybutyric acid and decreased serum bicarbonate and urine pH); a dose of ≥900 mg/kg/day resulted in moribundity/mortality and clinical signs of toxicity [33]. Although the therapeutical dose has been deeply studied, the exact mechanism of action for metformin is still not completely understood, but it is known that in various tissues and organs, it improves glucose metabolism via activation of the ubiquitously expressed AMP-activated protein kinase (AMPK) [17,34]. The AMPK is a Ser/Thr protein kinase that acts as a sensor of the cellular energy status and modulates metabolic pathways of carbohydrates and lipids via the inhibition of enzymes involved in gluconeogenesis and glycogen synthesis. Thus, overproduction of glucose from the liver is controlled by means of decreasing the phosphorylation of essential substrates for glucose output, reducing cAMP and glucagon action, as well as AMPK activation in a fasting state and inhibiting fatty acid synthesis, while mitochondrial oxidative phosphorylation is stimulated [35,36].

Therefore, due to a LOAEL dose of Cd causes metabolic disruptions such as insulin resistance, dyslipidemia, dysglycemia, and metabolic syndrome. The aim of this work was to investigate, in Wistar rats, the effect of a NOAEL dose of metformin on the homeostasis of carbohydrates and lipid in serum and tissues after a chronic Cd exposition.

## 2. Material and Methods

### 2.1. Animals and Treatments

One hundred male Wistar rats, weighing 70 to 80 g, obtained from the Claude Bernard vivarium of the Universidad Autónoma de Puebla, Mexico were housed in polycarbonate boxes with a sawdust bed and maintained under temperature-controlled (19–26 °C), 12-h light–dark cycles, with free access to food and water. The animals were conditioned with a standard diet until reaching 100 g. Once the rats reached this weight, they were randomly divided into two groups: “Control” (standard diet Labdiet 5001 and water *ad libitum*, *n* = 50) and “Cadmium” (standard 5001 Labdiet diet with drinking water containing 32.5 ppm of Cd *ad libitum*, *n* = 50). At the end of the third month, 10 “Control” rats and 10 rats from the “Cadmium” group were sacrificed to ensure the metabolic disruption. The cadmium group was then divided into two subgroups: “Cadmium” alone subgroup (*n* = 20), and the “Cd + Metformin” subgroup (standard 5001 Labdiet diet, drinking water containing 32.5 ppm of Cd *ad libitum* and Metformin treatment 200 mg/kg/day; oral via; *n* = 20). The control group also was divided into control (standard 5001 Labdiet diet, drinking water free Cd; *n* = 20) and Metformin (standard 5001 Labdiet diet, drinking water free Cd and Metformin treatment 200 mg/kg/day; oral via; *n* = 20) groups. All groups were kept under these conditions for two more months. The metformin dose used was chosen based on previous reports of a no observable adverse effect level (NOAEL) and the effective dose (ED_50_) as hypoglycemic and hypolipidemic [25,33]. Just prior to each cohort time (3, 4, and 5 months), the rats received an oral glucose load (TOG), equivalent to 1.75 g of glucose/kg weight. The rats were anesthetized intraperitoneally with xylazine/ketamine (20/137 mg/kg) and under anesthesia, whole blood (500 µL) was drawn via cardiac puncture at 0, 30, 60, and 90 min. The serum was then separated by centrifugation and stored at −70 °C, and after, tissues (liver, biceps femoris muscle, heart, kidney, and retroventral adipose tissue) were immediately removed and thoroughly perfused with cold saline and stored at −70 °C until the analysis. Each procedure was performed according to the National Institute of Health’s guide for the care and use of Laboratory Animals and the Guide for the Care and Use of Laboratory Animals of the Mexican Council for Animal Care NOM-062-ZOO-1999, European Convention for the Protection of Vertebrate Animals Used for Experimental and other Scientific Purposes, Guiding Principles in the Use of Animals in Toxicology, and it was approved by the Institutional Committee for the Care and Use of Animals on 10 November 2015. Every effort was made to minimize the number of animals used and to ensure minimal pain and/or discomfort.

### 2.2. Animal Zoometry

Weight, fat percentage, and size of the rats were monitored weekly. The weight was measured using a digital balance (Torrey, City of Mexico, State of MEX, Mexico; model: LPCR-20/40) and the size of each animal was obtained by measuring the length from the base of the tail to the tip of the nose. The abdomen diameter was estimated using the diaphragm zone as an upper limit and the fold of the legs as the bottom limit. The body mass index (BMI) was calculated using the formula weight/size^2^ and fat percentage was calculated according to the Lee index for rodent models, with the formula: % fat = [(weight in g ^(0.33)^)/size in cm] × 100 [37].

### 2.3. Biochemical Assays in Serum

From the serum obtained at time 0 min after 4–5 h fasting, the concentrations of glucose, lactate, total lipids, triglycerides, cholesterol, low-density lipoprotein cholesterol (LDL), and high-density lipoprotein cholesterol (HDL) were determined using spectrophotometry with commercial kits and an automatic analyzer AutoKemII (KONTROLab, Company, Morelia, MICH, Mexico). The level of very low-density lipoprotein (VLDL) was obtained using the Friedenwald equation [38]. Free fatty acid (FFA) concentration was determined according to the method described by Brunk and Swanson (1981), in a Perkin Elmer EZ150 model Lambda (Tres Cantos, MAD, Spain) spectrophotometer at 620 nm wavelength [39]. Lipoprotein sub-fractions were characterized using a polyacrylamide gel disc electrophoresis, as described by Rainwater et al. [40]. Three gradients of different pore size were prepared to allow for the separation of pre-beta (VLDL1 and VLDL2), beta (LDL I, II, IIIA, IIIB, IVA, and IVB) and alpha (HDL2a, 2b, 3a, 3b, and 3c) sub-fractions. To determine the different levels of lipoprotein sub-fractions, a densitometric analysis of the discs was performed in the polyacrylamide gel and then the area under the curve was quantified by using ImageJ software (National Institutes of Health, Bethesda, MD, USA).

### 2.4. Insulin Resistance Analysis

Plasma insulin concentrations were determined using an ELISA immunoassay (Diagnostica Internacional Company, Guadalajara, JAL, Mexico), with the resulting antibody–antigen complex assessed at 415 nm in a Stat fax 2600 plate reader (WinerLab Group, ROS, Argentina). Insulin resistance using homeostasis model assessment insulin resistance (HOMA-IR), insulin resistance adipocyte dysfunction (IDA-IR), and insulin sensitivity using hepatic insulin sensitivity (HIS) was evaluated using mathematical models according to the report by Treviño et al. [37].

### 2.5. Glycogen and Triglycerides Content in Tissues

Biopsies from tissues (liver, heart, renal cortex, renal medulla, and retroventral adipose) were homogenized at 100 mg in 800 μL of isotonic saline solution (ISS) to assess triglyceride content, whereas a second dilution was made only for adipose tissue, in which the homogenate was diluted 1:2 with ISS and the protocol for the triglyceride kit described by the manufacturer was followed. For the determination of glycogen, we followed the technique described by Bennett et al., from 150 mg of each tissue homogenized with 2 mL of perchloric acid [41].

### 2.6. Statistical Analysis

The results are expressed as a mean ± standard error of the mean (SEM) before beginning the metformin treatment (3 months). The statistical difference between the control and the cadmium group was determined by using a Student unpaired t-test with a significance level of *p* ≤ 0.05. Results obtained after 4 and 5 months of treatments were analyzed by using a one-way ANOVA test and Bonferroni post hoc test, considering *p* ≤ 0.05 as statistically significant.

## 3. Results

### 3.1. Morphometry, Lipids and Carbohydrates in Serum and Tissues after 3 Months of Cd Exposure

**Figure 1:**
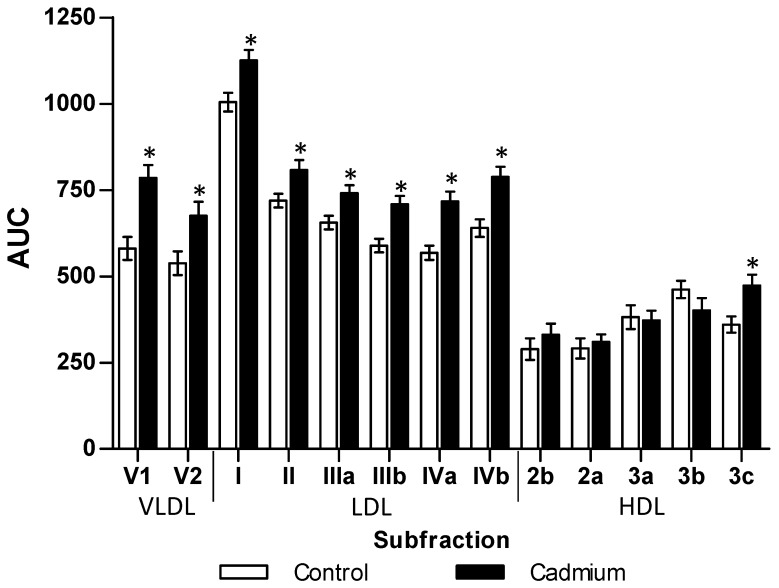
Disturbances caused by cadmium exposition on subfractions of lipoproteins. The results shown are the average of ten separate experimental animals ± SEM. (*) Indicates significant differences from the control group with *p* ≤ 0.05 using a Student *t*-test.

The chronic Cd exposure in a lowest observed adverse effect level dosage (LOAEL, 32.5 ppm) after 3 months produced zoometry modifications that increased weight (29%), abdominal perimeter (22%), body mass index (45%), and percentage of fat (15%). The lipid profile showed a similar result by significantly increasing total lipids (32%) and triglycerides (138%) in serum. Although the total cholesterol showed no difference, the VLDL and LDL fractions increased by 42% and 98%, respectively. Meanwhile, the HDL fraction showed a significant decrease of 45% (Table 1). The analysis of subfractions (Figure 1) showed an increase in V1 (35%) and V2 (26%), as well as LDL I, II, IIIa, IIIb, IVa, and IVb subfractions of 12%, 12%, 13%, 20%, 26%, and 23%, respectively. Exclusively, the HDL3c subfraction increased by 31% in comparison to the control group (Figure 1). Dyslipidemia from Cd exposure also affects triglycerides stored in different tissues, increasing significantly in the liver (39%), muscle (198%), heart (78%), renal cortex (112%), renal medulla (54%) and retroventral adipose (27%).

On the other hand, the carbohydrate homeostasis was also affected, fasting glucose and postprandial glucose after a load of 1.75 g/kg showed significant increases, which corresponded to 106%, 125% (30′ post-load), 193% (60′ post-load), and 210% (90′ post-load); likewise, the lactate level was augmented by 28%. However, in some tissues, glycogen deposits were significantly diminished: in the liver (36%), heart (37%), and renal cortex (53%), while the muscle showed an increase of 107%. The glycogen content in the renal medulla and retroventral adipose showed no difference (Table 1). The zoometric, metabolic, and biochemical changes observed in the rats of the Cd group were in concordance with significant hyperinsulinemia (75%), the development of insulin resistance demonstrated by HOMA-IR (216%), and insulin resistance adipocyte dysfunction (IDA-IR; 557%), as well as a significant loss of hepatic insulin sensitivity (HIS; 72%) (Table 1).

### 3.2. Metformin Treatment on the Metabolic Disruption Caused by Cd Exposure

**Figure 2:**
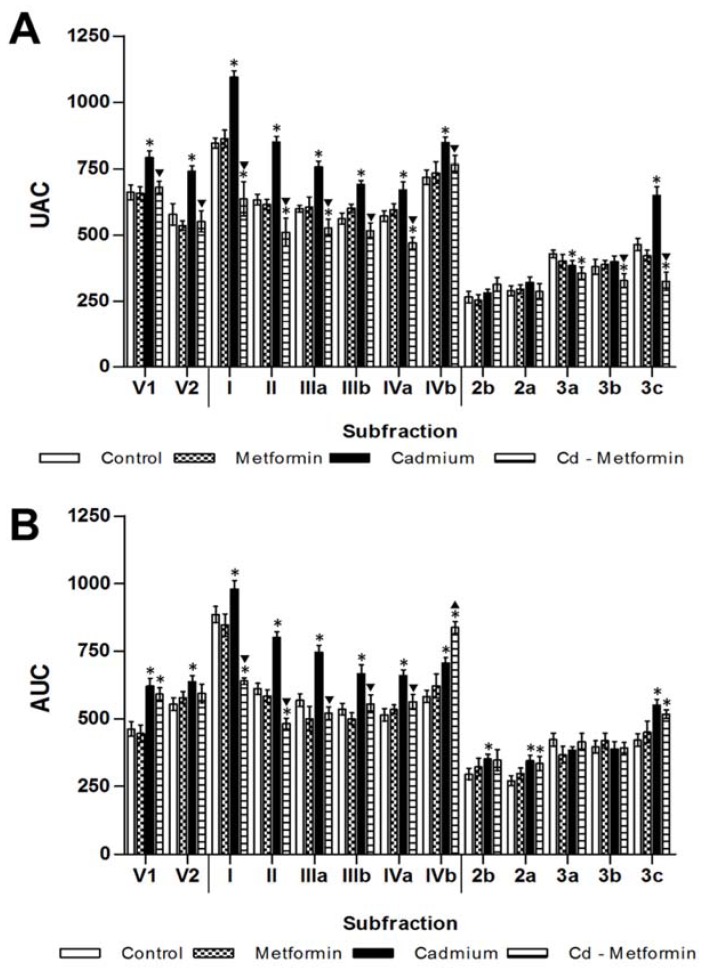
Metformin effect on lipoprotein subfractions. (**A**) One month with the different treatments. (**B**) Two months with the different treatments. The results shown are the average of ten separate experimental animals ± SEM. (*) Indicates significant differences from the control group. (▼) Indicates significant decreases with respect to the cadmium group. (▲) Indicates significant increases with respect to the cadmium group *p* ≤ 0.05 using an ANOVA test with a Bonferroni post hoc test.

**Figure 3:**
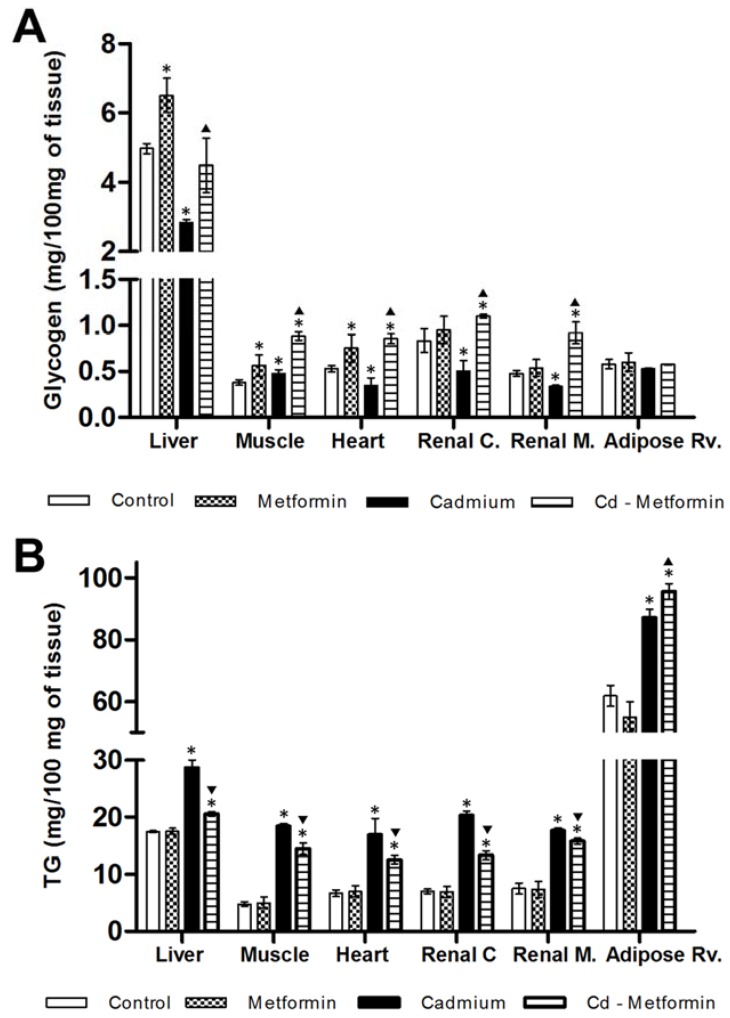
Glycogen and triglyceride concentration in tissues at one month with the different treatments: (**A**) glycogen, and (**B**) triglycerides. The results shown are the average of ten separate experimental animals ± SEM. (*) Indicates significant differences from the control group. (▼) Indicates significant decreases with respect to the cadmium group. (▲) Indicates significant increases with respect to the cadmium group *p* ≤ 0.05 using an ANOVA test with a Bonferroni post hoc test.

The metformin group after 1 and 2 months of administration did not show differences in the zoometry and serum parameters in relation to the control group. The Cd group remained altered after the fourth month of exposition in zoometric and biochemical parameters (Table 2). Meanwhile, the Cd + metformin group (1 month of treatment) showed an improvement of zoometric parameters, such as weight, abdominal perimeter, and BMI; however, there was now a complete regulation in the percentage of fat, which remained 6% above the control group (Table 2). The lipid biomarkers also showed a slight improvement without reaching the values of the control group, where total lipids, FFA, and triglycerides remained high at 24%, 79%, and 66%, respectively. However, the lipoproteins VLDL, LDL, and HDL improved. According to the total fractions, the subfractions of VLDL (V1 and V2) showed no differences compared with control group, while the subfractions of LDL I to IVa (25%, 20%, 12%, 8%, and 18%) and the HDL 3a–3c (17%, 14%, and 30%) (Figure 2) were significantly diminished. All subfractions of the Cd + metformin group showed improvement in relation to the Cd group. Triglycerides in the tissues of the Cd + metformin group showed a significant reduction compared to the Cd group, except for the adipose tissue. However, when the Cd + metformin group was compared with the control group, triglycerides remained high in the liver (18%), muscle (201%), heart (87%), renal cortex (89%), renal medulla (100%), and retroventral adipose tissue (55%) (Figure 3B). The metformin group did not show differences of stored triglycerides in tissues versus the control group.

In relation to the glucose homeostasis, Cd exposure affected the oral glucose tolerance and glycogen concentration while decreasing it in the tissues (except in muscle; Figure 3A). Metformin administration did not affect lactate level, the oral glucose tolerance and increased the glycogen level in the liver, muscle, and heart. Also, metformin co-administered with Cd mild improved the oral glucose tolerance. In this regard, lactate and fasting glucose did not show differences in relation to the control group (Table 2), although glucose remained elevated postprandially, with 44% (30′ post-load), 77% (60′ post-load), and 75% (90′ post-load). Moreover, the glycogen level in the liver and retroventral adipose tissue was no different in comparison with the control group but was higher in muscles (134%), heart (60%), renal cortex (32%), and renal medulla (92%) (Figure 3A). Compared to the control group, both the Cd group (90%) and the Cd + metformin group (170%) presented hyperinsulinemia, although, in the Cd + metformin group, HOMA-IR and ADA-IR improved, although not as much as in the control group, and HIS remained the same (Table 2).

**Figure 4:**
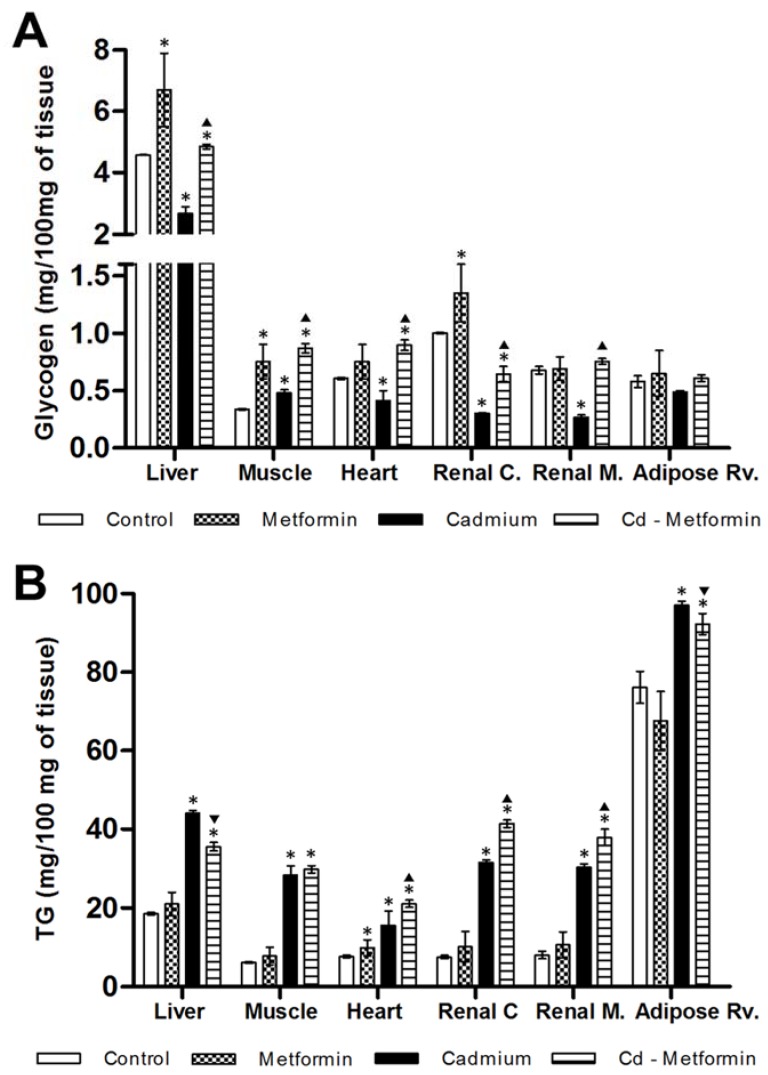
Metformin effect on lipoprotein subfractions. (**A**) Two months with the different treatments. (**B**) Two months with the different treatments. The results shown are the average of 10 separate experimental animals ± SEM. (*) Indicates significant differences with values above the control group. (▼) Indicates significant decreases with respect to the cadmium group. (▲) Indicates significant increases with respect to the cadmium group *p* ≤ 0.05 using an ANOVA test with a Bonferroni post hoc test.

After five months, rats exposed to Cd presented a greater metabolic disorder, showing evidence of zoometric worsening as well as lipid and glucose homeostasis in both serum and tissues, with a marked development of insulin resistance and loss of hepatic insulin sensitivity. In contrast, animals exposed to Cd and treated with metformin for two months showed zoometric parameters like the control group, except for the percentage of the body fat (5% greater). The panel of lipids showed incomplete recovery because the levels of total lipids remained increased (44%), FFA (69%), triglyceride (125%), and VLDL (141%), while HDL remained low (35%) (Table 3). Cholesterol subfractions of the metformin-treated group exhibited levels like the control group in V2 but not in V1 (28%, high), in LDL I to IVa but not in IVb (44%, high), as well as HDL2b, 3a, and 3b, but not in 2a and 3c (24% and 23%, high) (Figure 2B). The triglyceride deposits in rats administrated with metformin alone showed a tendency to increase; in this group, the heart observed a significant difference. With regard to the group co-treated with Cd and metformin, it showed overstoring in the liver (91%), in the muscle (384%), heart (176%), and renal cortex and medulla (451% and 369%), and retroventral adipose (21%) (Figure 4B).

Regarding glycogen, the metformin treatment itself increased levels in the liver, muscle, renal cortex, and heart, but the heart increase was not significant. Meanwhile, in rats Cd-exposed co-administered with metformin glycogen improved the content in the liver (6%), muscle (157%), heart (48%), and renal medulla (12%), but not in the renal cortex, where it diminished (36%) (Figure 4A). Likewise, lactate and the oral glucose tolerance showed improvement after metformin treatment, although it remained slightly elevated (0′, 17%; 30′, 16%; 90′, 19%; 90′, 29%). Hyperinsulinemia was also observed (216%), in concordance with insulin resistance by HOMA (157%) and IDA-IR (2450%), both greater than the control group, while hepatic insulin sensitivity fell (73%) (Table 3).

## 4. Discussion

In this paper, we studied the role of metformin after a metabolic disruption caused by exposure to Cd. Previously, we demonstrated that a LOAEL dose of Cd in drinking water produces a metabolic toxicity that is characterized by insulin resistance in multiple peripheral tissues, increasing insulin release with hyperglycemia and lipid metabolism alterations [37]. Although Cd toxicology has been extensively discussed, associating its toxic effects with inflammation, oxidative stress, and genotoxicity processes [2,42], the metabolic toxicity is not considered, and thus, the associated mechanism is poorly studied. The literature is somewhat contradictory in relation to Cd exposure and metabolic complications in lipids, glucose, overweight, obesity, and diabetes [43,44,45,46]. The results obtained in this work show clearly that a chronic exposition to a Cd LOAEL dose produces the metabolic disorders mentioned above. In addition to the metabolic changes, our results demonstrate new evidence of the progressive accumulation of triglycerides in different tissues and disturbances on glycogen deposits.

In humans, exposure to Cd has been related to hyperglycemia, impaired fasting glucose, and a positive correlation in patients with type 2 diabetes mellitus in a dose-dependent manner related to the kind and time of exposure [47,48,49]. In animal models, the hyperglycemia produced by Cd has been related with significant increases in the hepatic transporter GLUT2, carbohydrate regulatory element binding protein (ChREBP), and mRNAs of glucokinase and pyruvate kinase [50], as well as by a downregulation of the expression of the glucose transporter GLUT4 in both muscle and adipocytes. These changes lead to a limited glycolysis, increases in the glycogenolysis, and the enzymatic activation of the gluconeogenesis pathway, which could explain the lactate increase and the poor glycogen storage observed in this and other works with rats exposed to cadmium [51,52,53,54,55]. It is well known that hyperglycemia is normally compensated with hyperinsulinemia as an adaptive response from the pancreas to restore glucose homeostasis, which is often linked to progressive insulin resistance, a key factor in the feedback between the liver and adipose tissue in relation to triglyceride storage. However, a limited number of works have shown the insulin resistance in relation to Cd exposure [37,56,57].

Hepatic insulin resistance, or low insulin sensitivity, favors lipogenesis, and thus the increase of novo synthesis of triglycerides and a higher secretion of VLDL type V1 and V2 (triglyceride-rich lipoproteins, TRLs), as was observed in our results [57,58,59]. VLDL carry triglycerides, increasing its accumulation in peripheral tissues (Table 1). Mechanisms associated with insulin resistance in adipose tissue, provoke an erroneous triglyceride storage, and thus an over-flux of FFA toward all peripheral tissues, the liver being the most affected such that more VLDL of type V1 is synthesized. These events produce a redundant cycle in which high levels of TLR and FFA in serum can be observed. TRLs induce small LDL formation that could contribute to atherosclerosis development and cardiovascular disease. Small LDL subfractions, corresponding to LDL-IIIa, IIIb, IVa, and IVb, are increased in the Cd-exposed rats [60,61,62]. TLRs could also alter HDL subfractions, where the HDL3c subfraction is particularly susceptible to Cd exposure. Under normal conditions, HDL3c acts as an anti-oxidant against LDL oxidation and has anti-thrombotic, anti-inflammatory, and anti-apoptotic activity; however, during insulin resistance, the small HDL formation is promoted, producing a triglyceride-rich HDL3c subfraction, thus modifying its activity [63,64,65]. Complementarily, it must also be considered that Cd accumulation could be a decisive factor in metabolic toxicity. In this regard, the cadmium ion (Cd^2+^) gets into the cells by different transporters such as the type 1 divalent metal transporter (DMT-1), zinc importer proteins (ZIP’s), and voltage-gated calcium channels (VGCC) [3]. It has recently been demonstrated that not only does Cd^2+^ act as a substrate for the organic cation transporter 2 (OCT2) in a dose-dependent manner, but it also upregulates its expression and maximum transport rate (V_max_), which might serve as a mechanism for Cd accumulation [10,66].

On the other hand, once Cd has caused a metabolic disruption, we began metformin treatment under the hypothesis that the drug is effective in metabolic control in a NOAEL dose (based on the mortality, biochemical, and body weight effects), regardless of the origin of the metabolic dysregulation, for example, hypercaloric diets consumption, energetic imbalances, neuroendocrinal disorders, and pharmacological and no-pharmacological supplementation, or in this case, by cadmium exposition. The metformin administration in rats without metabolic disorders does not show important biochemical changes in serum, but the drug positively modified the glycogen stored, as will be discussed later. In rats exposed to Cd and treated with a NOAEL dose of metformin, blood glucose levels are lowered and insulin resistance is ameliorated. Moreover, metformin elicits additional benefits, including improvement of lipid profiles, prevention of vascular complications, and lowering of the potential for hypoglycemia. Considering that metformin uses organic cation transporters (OCT1, OCT2, and OCT3) as an influx into the cells [29,30]. Rats that received one and two months of metformin treatment, including Cd exposure, showed a reduction in weight, abdominal perimeter, BMI, and percentage of body fat (Table 2 and Table 3), which are results that were consistent with other works in both humans and Wistar rats that present metabolic complications by means other different to cadmium exposure [25,36]. These findings agree with the fact that metformin increases the AMP-activated protein kinase (AMPK), which is a “metabolic master switch”; its activation inhibits energy-consuming pathways and stimulates ATP-producing catabolic pathways [17]. In addition, AMPK activation can inhibit fatty acid synthesis by inhibition of acetyl-CoA carboxylase 1 and 2 and malonyl-CoA content reduction [17,67]. In this regard, a NOAEL dose of metformin is not completely effective because tissues showed a mild decrease in triglyceride storage after one month. However, in the second month, in tissues such as muscle, heart, and kidney, the triglyceride content increased (steatosis multi-tissue), even more than for the Cd group, although the liver observed a minimal recovery. The steatosis is a phenomenon common that is observed in an excess of metabolic needs or a limited rate of energy obtained via lipids (low lipid oxidation). Also, steatosis can generate lipotoxicity by lipotoxic intermediates, such as ceramide and acylcarnitine, and is promoted by triglyceride mobilization into VLDL (Figure 3 and Figure 4), as shown by the lipid profile.

In relation to the serum lipid profile in rats exposed to Cd, after the treatment with metformin, a moderate improvement was observed in triglycerides, cholesterol, and the VLDL, LDL, and HDL fractions, but not at the level of the control group (Table 2 and Table 3). Changes in cholesterol fractions also imply modifications of each subfraction (Figure 2). We observed that metformin had a limited action on lipid regulation because only the first month of treatment showed regulation, but in the second month, V1 and V2 subfractions followed the same pattern as the Cd group, suggesting a liver response to process lipidic clearance. However, almost all LDL subfractions were maintained in the control group, except for LDL-IVb, which is considered small, dense, and highly atherogenic [62]. It is interesting that the co-administration of cadmium and metformin treatment over-increases only this subfraction. The HDL showed a similar pattern because large and small subfractions were regulated, except for HDL3c, which is related to a poor glycemic control and an increase in the atherogenic risk [64]. The lipoprotein improvement is attributable to a lower insulin resistance and an enhancement of hepatic sensibility that is well recognized in metformin treatment [29,30,68,69]. However, in the presence of Cd, the time of metformin administration was probably insufficient because the hepatic sensibility was not completely restored, and the resistance was not eradicated, which explains the high FFA level coming from adipose tissue.

Additionally, a lower blood glucose was observed in both fasting and 1.75 g post-load of glucose/kg (Table 2 and Table 3). Several mechanisms have been proposed to explain this action as a reduction of hepatic gluconeogenesis mediated by AMPK activation that inhibits the PKA pathway, diminishes the hepatic uptake of gluconeogenic substrates, and activates glycolysis [70,71,72]. At the muscle level, AMPK activation can also increase glucose consumption, optimizing energy expenditure and production, and participates in the transition glycolytic to oxidation of fatty acids [17]. In addition, the metformin treatment can improve the insulin action on the glycogenic pathway, as was observed in both groups, the metformin alone and Cd-metformin co-treated groups, in which glycogen concentration increased even more than the control group in almost all tissues analyzed [73,74]. Although the role of gluconeogenesis as a source of hepatic glucose overproduction and as a target of metformin action are well described, less is known about the role of changes in glycogen. However, our results suggest an increased activity of the glycogenic enzyme phosphoglucomutase and the decreased activity of glycogenolytic enzyme glycogen phosphorylase by the treatment, because glycogen phosphorylase is a rate-limiting enzyme of glycogenolysis and is regulated by phosphorylation and by the allosteric binding of AMP, ATP, glucose-6-phosphate, and glucose. Additionally, an effect observed in the group co-administered with Cd and metformin had a lactate increase (Table 1 and Table 2). This was probably the result of an activity decrease of glucose 6-phosphatase and glucose 6-phosphate dehydrogenase, which had as a consequence an uncoupling of oxidative phosphorylation and the cycle of Krebs, which suppresses the lactate uptake, generating a high hepatic lactate production. This is a strong indicator of a low uptake of postprandial glucose [75,76,77]. The therapeutic doses of metformin usually cause little to no increase in basal and postprandial blood lactate levels (less than 1–2 mmol/L) but impair the hepatic metabolism. Also, a poor renal function by Cd accumulation associated with a metabolic kidney disruption could reduce lactate clearance [28].

Another important finding with the co-administration of Cd and metformin was hyperinsulinemia (Table 1 and Table 2). It is recognized that metformin helps to restore the response to insulin, but not in insulin secretion [29,30]. However, the rats exposed to Cd develop an impaired insulinemic response. Some mechanisms for this have been proposed, such as insulin receptor impairment, low insulin receptor affinity by occupancy or negative cooperation, and a reduction in the number of receptors. Our results strongly suggest that Cd sensitize to the β-cells, producing a sustained hyperfunction and metformin would act on non-canonical pathways leading to the high insulin secretion [37]. It is probable that Cd exposure additional to metformin administration alters the ATP/ADP ratio that is permissive for K channel closure and enhanced insulin secretion being more susceptible in the presence of higher glucose concentration at the basal and postprandial [78]. An elevation in the total cellular NADH/NAD ratio also has been shown to promote insulin exocytosis [79]. The insulin release is correlated with an increase of intracellular Ca^2+^, in which Cd^+2^ has been correlated. Changes in ΔΨ_m_ are promoted by both glucose and metformin, which also met the set criteria as a potential factor important in insulin release [80].

In summary, is very important to consider the origin and duration of metabolic disruption for therapeutic management because Cd exposure has demonstrated metabolic toxicity in carbohydrates and lipids pathways, as well as serious alterations of insulin resistance in multiple tissues. Therefore, the treatment must consider intracell disorders, such as modifications in glycogen and triglycerides storage, as well as dysglycemia and dyslipidemia, particularly in subfractions of small LDL and HDL. In this sense, the treatment with a NOAEL dose of metformin in co-administration with Cd was limited with regard to metabolic regulation, and in the chronicity, which was counter-productive in relation to lipids storage in non-adipose tissue. However, the increase in dosage could bring unexpected consequences, such as morbidity, mortality, and clinical signs of toxicity, in addition to increasing metabolic acidosis (due to lactate and beta-hydroxybutyric acid). Although a NOAEL metformin dose was more effective in the carbohydrates’ homeostasis, the associated metabolic pathways must be further studied and understood for establishing the therapeutic management in relation to the dosage and time of administration, selected on the basis of the metabolic disruption origin because the dose used in this work was demonstrated to be efficient in metabolic disorders from hypercaloric diet consumption, but not in cadmium exposition. Finally, the dosage selected must prevent clinical, metabolic, and toxicological complications since metformin is the first line of treatment for diabetes, obesity, overweight, insulin resistance, and other metabolic complications.

## Figures and Tables

**Table 1:** Metabolic disturbances caused by cadmium exposure.

Measurements	Control	Cadmium	Metabolite mg/100 mg of Tissue	Control	Cadmium
	*n* = 30	*n* = 50		*n* = 10	*n* = 10
**Morphometric panel:**			**Triglycerides:**		
Weight (g)	341 ± 3.4	439.2 ± 8.1 *	Liver	16.2 ± 0.7	22.5 ± 1 *
Abdominal perimeter (cm)	18.4 ± 0.1	22.4 ± 0.2 *	Muscle	3.36 ± 0.2	10.0 ± 0.3 *
Body mass index	0.93 ± 0.02	1.35 ± 0.05 *	Heart	5.06 ± 0.3	9 ± 0.2 *
% Body fat	35.9 ± 0.1	41.3 ± 0.5 *	Renal cortex	5.98 ± 0.4	12.7 ± 0.6 *
**Lipidic panel (mg/dL):**			Renal medulla	7.46 ± 0.3	11.5 ± 0.7 *
Total lipids	184 ± 12	243 ± 5.1 *	Rv Adipose	51.2 ± 1.4	64.8 ± 1.6 *
FFA	2.18 ± 0.03	5.18 ± 1.1 *			
Triglycerides	64.4 ± 2.5	106.8 ± 3.1 *	**Glycogen:**		
Total Cholesterol	103.1 ± 7.5	103.4 ± 6	Liver	4.2 ± 0.4	2.9 ± 0.3 *
**Cholesterol fraction:**			Muscle	0.3 ± 0.03	0.62 ± 0.1 *
VLDL	13.5 ± 1.5	19.2 ± 1.2 *	Heart	0.9 ± 0.05	0.57 ± 0.8 *
LDL	24.2 ± 4	47.9 ± 3.1 *	Renal cortex	1.2 ± 0.2	0.57 ± 0.11 *
HDL	65.4 ± 2	36.3 ± 1.5 *	Renal medulla	0.56 ± 0.2	0.51 ± 0.6
**Carbohydrate panel:**			Rv. Adipose	0.55 ± 0.03	0.48 ± 0.01
Lactate (mmol/L)	7.3 ± 0.7	9.4 ± 0.5 *	**Insulin resistance panel:**		
Fasting glucose (mg/dL)	80 ± 3.2	165 ± 5 *	Insulin (µUI/mL)	12 ± 3.1	21 ± 4.5 *
‡ Glucose 30 min (mg/dL)	107.1 ± 2.9	241 ± 8.1 *	HOMA-IR	0.44 ± 0.05	1.39 ± 0.19 *
‡ Glucose 60 min (mg/dL)	90.6 ± 3.4	265 ± 7 *	IDA-IR	0.07 ± 0.03	0.46 ± 0.15 *
‡ Glucose 90 min (mg/dL)	81.2 ± 3	251.5 ± 4.2 *	HIS	18.8 ± 3.5	5.2 ± 2.2 *

The biochemical and morphometric results shown are the average of 80 separate experimental animals ± SEM. Meanwhile, the metabolite/100 mg of tissue results shown are 20 separate experimental animals ± SEM. (*) Indicates significant differences from the control group with *p* ≤ 0.05 using a Student *t*-test. (‡) values obtained after a load glucose 1.75 g/kg. FFA = free fatty acid; VLDL = very low-density lipoprotein; LDL = low-density lipoprotein; HDL = high-density lipoprotein; Rv Adipose = retroventral adipose. HOMA-IR = homeostasis model assessment insulin resistance; IDA-IR = Insulin resistance adipocyte dysfunction; HIS = Hepatic insulin sensitivity.

**Table 2:** Zoometric and metabolic evaluation after 1 month of metformin treatment.

Measurements	Control	Metformin	Cadmium	Cd + Metformin
	*n* = 10	*n* = 10	*n* = 10	*n* = 10
**Morphometric panel:**				
Weight (g)	401.6 ± 7.9	380.2 ±15.3	470 ± 6.3 *	418 ± 10 ▼
Abdominal perimeter (cm)	20.8 ± 0.1	19.4 ± 0.6	24.2 ± 0.7 *	21.1 ± 0.4 ▼
Body mass index	1.1 ± 0.04	1.0 ± 0.02	1.4 ± 0.01 *	1.1 ± 0.03 ▼
% Body fat	37.8 ± 0.3	35.7 ± 1.6	42.1 ± 0.1 *	40.0 ± 0.3 *▼
**Lipid panel (mg/dL):**				
Total lipids	187.2 ± 9.6	180.1 ± 3.3	253.6 ± 4.5 *	232 ± 6.1 *▼
FFA	2.85 ± 0.2	3.01 ± 0.2	6.49 ± 0.1 *	5.1 ± 0.1 *▼
Triglycerides	56 ± 6	51 ± 2	112.3 ± 4.5 *	93.1 ± 3.5 *▼
Total Cholesterol	111.9 ± 5.6	107.9 ± 4.9	108.1 ± 6.7	84.9 ± 5.7 * ▼
Cholesterol fraction				
VLDL	16 ± 1.6	18 ± 0.9	21.2 ± 0.9 *	18.2 ± 1 *▼
LDL	36 ± 3.5	40 ± 6.1	54.8 ± 1.7 *	26 ± 2.1 *▼
HDL	59.9 ± 2.6	49.9 ± 7.2	32.1 ± 2.5 *	40.7 ± 3.1 *▲
**Carbohydrate panel:**				
Lactate (mmol/L)	7.3 ± 0.7	7.5 ± 0.2	8.55 ± 0.3 *	8.2 ± 0.1 ▼
Fasting glucose (mg/dL)	80.0 ± 6.3	76.0 ± 4.8	135 ± 4.1 *	79.7 ± 4.3 ▼
Glucose 30 min (mg/dL)	107.1 ± 3.8	97.7 ± 5.2	238 ± 6.7 *	154.4 ± 5.4 *▼
Glucose 60 min (mg/dL)	90.6 ± 4.3	88.4 ± 7.1	250 ± 9.4 *	160.4 ± 3.8 *▼
Glucose 90 min (mg/dL)	81.2 ± 5.0	73.9 ± 9.4	235 ± 8.8 *	142.2 ± 6.1 *▼
**Insulin resistance panel:**				
Insulin (µUI/mL)	10 ± 3.4	9.8 ± 4.8	19 ± 2.4 *	27 ± 4.9 *
HOMA-IR	0.44 ± 0.03	0.40 ± 0.08	1.42 ± 0.19 *	0.88 ± 0.21 *▼
IDA-IR	0.02 ± 0.02	0.02 ± 0.01	0.54 ± 0.12 *	0.35 ± 0.11 *
HIS	22.5 ± 5.19	20.1 ± 3.3	7.01 ± 3.8 *	8.3 ± 2.2 *

The results shown are the average of 10 separate experimental animals ± SEM. (*) Indicates significant differences from the control group *p* ≤ 0.05. (▼) Indicate significant decreases with respect to the cadmium group. (▲) indicates significant increases with respect to the cadmium group *p* ≤ 0.05 using a one-way ANOVA test with a Bonferroni post hoc test.

**Table 3:** Zoometric and metabolic evaluation after 2 months of metformin treatment.

Measurements	Control	Metformin	Cadmium	Cd + Metformin
	*n* = 10	*n* = 10	*n* = 10	*n* = 10
**Morphometric panel:**				
Weight (g)	434.3 ± 6.2	420.9 ± 12.8	502.6 ± 9.5 *	442.6 ± 10.5 ▼
Abdominal perimeter (cm)	22.1 ± 0.6	20.7 ± 0.9	26.2 ± 0.5 *	22.9 ± 0.6 ▼
Body mass index	1.1 ± 0.04	1.0 ± 0.08	1.5 ± 0.03 *	1.2 ± 0.03 ▼
% Body fat	38.8 ± 0.3	36.2 ± 1.1	43.1 ± 0.1 *	40.8 ± 0.3 *▼
**Lipid panel (mg/dL):**				
Total lipids	180 ± 13	178 ± 9	270 ± 6.1 *	259 ± 4.5 *
FFA	3.49 ± 0.12	3.98 ± 0.6	6.8 ± 0.16 *	5.9 ± 0.2 *▼
Triglycerides	50.5 ± 5.8	47.7 ± 5.1	121.7 ± 2.1 *	113.6 ± 3.1 *▼
Total Cholesterol	102.2 ± 10.8	98.5 ± 6.7	112.9 ± 5.2	102.6 ± 3.2
Cholesterol fraction				
VLDL	10.1 ± 1.8	10.1 ± 1.8	24.9 ± 1.2 *	24.3 ± 1.2 *
LDL	39 ± 7	41 ± 3	59.5 ± 2.7 *	43.5 ± 4.7 ▼
HDL	53.1 ± 2	50.9 ± 3	28.5 ± 2.4 *	34.8 ± 1.4 *▲
**Carbohydrate panel:**				
Lactate (mmol/L)	8.2 ± 0.5	9.1 ± 1.3	11.3 ± 0.4 *	10.1 ± 0.3 *▼
Fasting glucose (mg/dL)	72.9 ± 4	67.7 ± 7	139.5 ± 4.2 *	85 ± 5.9 *▼
Glucose 30 min (mg/dL)	93.1 ± 0.6	85.3 ± 4.9	157.4 ± 14.4 *	108.3 ± 6.0 *▼
Glucose 60 min (mg/dL)	98.2 ± 3.0	104.4 ± 5.5	171.3 ± 8.7 *	117.0 ± 5.3 *▼
Glucose 90 min (mg/dL)	90.0 ± 5.0	98.0 ± 7.3	164 ± 6.3 *	116.2 ± 4.8 *▼
**Insulin resistance panel:**				
Insulin (µUI/mL)	11 ± 1.8	14 ± 2.9	25 ± 3.2 *	34.7 ± 4.1 *▲
HOMA-IR	0.47 ± 0.03	0.50 ± 0.1	1.69 ± 0.2 *	1.21 ± 0.23 *
IDA-IR	0.02 ± 0.01	0.03 ± 0.01	0.63 ± 0.17 *	0.51 ± 0.2 *
HIS	22.4 ± 4.7	24.7 ± 5.1	6.02 ± 1.9 *	6.10 ± 2.1 *

The results shown are the average of 10 separate experimental animals ± SEM. (*) Indicates significant differences from the control group *p* ≤ 0.05. (▼) Indicates significant decreases with respect to the cadmium group. (▲) Indicates significant increases with respect to the cadmium group *p* ≤ 0.05 using a one-way ANOVA test with a Bonferroni post hoc test.
